# A die is cast: autologous neutralizing antibody resistance shapes the HIV reservoir during uninterrupted ART

**DOI:** 10.1172/JCI198353

**Published:** 2025-10-01

**Authors:** Nancie M. Archin

**Affiliations:** 1 UNC HIV Cure Center, and; 2 Department of Medicine, The University of North Carolina at Chapel Hill, Chapel Hill, North Carolina, USA.

## Abstract

Initial efforts to control HIV infection include an autologous neutralizing antibody (aNAb) response. aNAbs bind Env trimers of the infecting HIV strain to neutralize virus but are not very effective at controlling HIV, as the virus quickly develops escape mutations to evade neutralization. Nevertheless, recent evidence suggests that aNAbs exert ongoing immune pressure on viral isolates in people living with HIV (PWH) treated with anti-retroviral therapy (ART) during chronic and early infection. In this issue of the JCI, McMyn et al. studied the dynamics of aNAb resistance in a cohort of 31 PWH treated with ART. Notably, a large proportion of HIV reservoir viral isolates were resistant to aNAb neutralization, which correlated with longer duration on uninterrupted ART, suggesting that selection for aNAb-resistant isolates occurs as reservoir cells containing neutralization-sensitive isolates are eliminated. aNAb resistance was not attributed to waning antibody response, which persisted for over 20 years despite viral suppression.

## Neutralizing antibodies and HIV infection

The humoral immune response to HIV surface envelope (Env) glycoprotein is complex. During primary infection, individuals produce high titers of antibodies targeting various epitopes (small antigen regions) of the Env glycoproteins, gp120 and gp41. During the first 2–4 weeks of infection, most of the antibodies against HIV are nonneutralizing. These include antibodies made against epitopes that are not presented in the native Env trimer spike, a subset of which still possess antiviral activity through Fc-mediated activity on destabilized Env trimers. It takes about 2–3 months of continuing virus replication for antibodies that can neutralize HIV to be detected in some individuals (1, 2). Neutralization is defined as the ability of antibodies to bind virions and prevent subsequent infection of host cells. In the case of HIV, most of the early neutralizing antibodies are autologous (aNAbs) in that they are viral-strain specific. These aNAbs target epitopes on the variable (V) loops and other regions of gp120 that are unique to the evolution of HIV variants in a single individual. Moreover, ongoing mutations in the variable Env epitopes targeted by aNAbs, which occur during reverse transcription of replicating viruses, allow continuous escape from their neutralization (3). Furthermore, HIV Env is heavily glycosylated, and mutations in Env can alter this glycan shield, aiding in evasion of neutralizing antibody response. About 15%–20% of people living with HIV (PWH) develop antibodies capable of neutralizing a wide variety of HIV strains during prolonged replication (4). These broadly neutralizing antibodies (bNAbs) tend to target conserved epitopes in Env and take years to evolve. Over many years of infection, about 1% of PWH develop bNAbs with exceptional potency and breadth of neutralization across the majority of HIV strains; this population is referred to as elite neutralizers (4).

The early establishment of a stable HIV reservoir remains a prominent barrier to an HIV cure. This persistent reservoir is best characterized in CD4^+^ T cells. Given their ability to target and neutralize a broad spectrum of HIV strains, administration of nonautologous bNAbs synthesized ex vivo are currently being explored as not only a method to suppress viral infection, but also as an avenue toward achieving an HIV cure through combination with other strategies such as latency reversal agents (5). However, several recent studies combining nonautologous bNAbs and latency reversal agents have had limited success in depleting the persistent HIV reservoir in humans (6, 7). Interestingly, despite the ineffectiveness of aNAbs in controlling HIV infection stemming from the rapid evolution of escape variants, aNAb-resistant HIV variants are present during episodes of virus rebound in PWH who started ART during chronic infection (8). This observation indicates ongoing immune pressure from aNAbs during chronic HIV infection and was further corroborated by several recent reports. In clinical studies using bNAb infusions to control HIV, it was observed that viruses emerging during analytical treatment interruption (ATI) were mostly distinct from those found in the peripheral reservoir (9). Extending our understanding of this phenomenon, Bertagnolli et al. demonstrated that in PWH treated during chronic infection, virus outgrowth performed in the presence of aNAbs resulted in selection of neutralization-resistant variants that were closely related to virus in ATI-rebound pool (10). More recently, analysis of pre-ART and post-ATI plasma samples of PWH who initiated ART early following infection showed that aNAb response appeared to mature while individuals were on suppressive therapy, indicating that the HIV epitope presentation occurred during suppressive ART. Furthermore, it was observed that HIV variants rebounding during an ATI were more resistant to contemporaneous autologous plasma neutralization compared with pre-ATI variants, highlighting the role of aNAbs in shaping which variants contribute to virus rebound (11).

## Digging further into aNAb resistance in the reservoir

In this issue of the JCI, McMyn et al. profiled neutralizing activity of contemporaneous aNAbs against inducible, replication-competent reservoir virus isolates obtained from quantitative viral outgrowth assays that were performed with CD4^+^ T cells obtained from 31 PWH who initiated ART during chronic infection (12). Five of these participants had previously experienced treatment interruption, either as part of a bNAb study (*n =* 3, ATI) or because of nonadherence to ART (*n =* 2). However, all were stably suppressed at the time of sample analysis. Env sequences for virus isolates were cloned into expression vectors to generate pseudovirus vectors used in neutralization assays. An inhibitory concentration (IC_50_) of more than 100 μg/mL was established as the cut off to classify isolates as resistant to neutralization. Among participants, there was substantial variation ranging from 0%–100% in the fraction of resistant isolates, but overall, a median of 92% resistant virus per participant was observed when analyzing both clonal and distinct viral isolates. In greater than 40% of PWH in the cohort, 100% of isolates were resistant. Interestingly, this analysis revealed a clear separation of aNAb resistance: in 20 participants, anywhere from 67%–100% of an individual’s viral isolates demonstrated aNAb resistance, and in 8 participants, between 0%–26% of an individual’s viral isolates exhibited aNAb resistance, meaning most of the viral isolates in this latter group were aNAb sensitive.

In trying to understand factors that may contribute to high or low percentages of aNAb-resistant isolates, McMyn et al. observed that average time on uninterrupted ART was significantly higher in the aNAb-resistant group compared with the aNAb-sensitive group. Correlation analysis of aNAb IC_50_ values or percentage resistance per person and time on ART (or time on uninterrupted ART) further demonstrated that longer duration on ART was associated with increased aNAb resistance, suggesting that perhaps, over time, aNAb-sensitive reservoirs are gradually eliminated. Detection of aNAb resistance in the latent reservoir was detected whether inducible, replication competent virus or proviral DNA were examined. McMyn et al. cleverly employed pharmacodynamics methods that are used to determine inhibition produced by antiviral agents and extrapolated the dose-response curves to in vivo aNAb concentrations. Interestingly, their results indicated that most of the viral isolates in the aNAb-resistant group were only weakly inhibited, and most isolates in the aNAb-sensitive group were inhibited at concentrations akin to the use of a single antiretroviral drug, highlighting the vulnerability of the aNAb response to evolution of escape variants. Only four isolates were inhibited by aNAbs at concentrations that were comparable to effective combination antiviral therapy. This suggests that aNAbs can inhibit replication in vivo, but they are no better than single-drug therapy and readily allow for the development of escape variants. Remarkably, however, most of the viral isolates from the aNAb-resistant group were effectively neutralized by at least one of the clinically relevant nonautologous bNAbs tested: VRC01, 10-1074, and PGDM1400.

An important finding of this study is that aNAbs persisted in most PWH even after 20 years of ART, and this was independent of reservoir size. By analyzing longitudinal plasma samples spanning many decades from three participants, McMyn and colleagues determined that aNAb activities varied depending on the participant or viral isolates tested, either remaining the same, improving, or waning. The number of participants analyzed longitudinally is small, which is a limitation. However, these preliminary results suggest a scenario in which as aNAb-sensitive viruses are eliminated, perhaps partially by NK cells through Antibody-Dependent Cell-Mediated Cytotoxicity (ADCC, as observed in vitro in this study), aNAb-resistant viruses persist in the reservoir and contribute to viral rebound in the absence of therapy.

## Implications and conclusions

In this carefully executed, important study, McMyn et al. highlight the profound stability of aNAbs, define factors that might contribute to the development of aNAb resistance in the reservoir, and provide context for the importance of aNAbs in eliminating reservoir viruses. The study provides further evidence that aNAb-resistant viral isolates may contribute to the rebound virus pool in the absence of therapy. It will be important to determine the best approach to eliminate aNAb-resistant isolates from the reservoir pool. In vitro experiments demonstrating that most of the resistant strains were susceptible to bNAb neutralization are encouraging. However, it is not clear why these variants are then not cleared by bNab therapy and invariably rebound in the absence of ART. Part of the reason may be limited target antigen expression, which reduces ADCC potency. It is also possible that more potent bNAbs or the administration of combination bNAbs during ATI are necessary to eliminate cells harboring aNAb-resistant virus. Lastly, with the widening interest in a personalized HIV cure, one wonders if aNAbs can be evolved ex vivo and exploited towards such an endeavor, perhaps by engineering autologous B cells using gene-editing technology to produce aNAbs directed towards dominant resistant epitopes in virus isolates identified in a given individual’s reservoir.

## Version 1 10/01/2025

Electronic publication
